# Multi-Class Clustering of Cancer Subtypes through SVM Based Ensemble of Pareto-Optimal Solutions for Gene Marker Identification

**DOI:** 10.1371/journal.pone.0013803

**Published:** 2010-11-12

**Authors:** Anirban Mukhopadhyay, Sanghamitra Bandyopadhyay, Ujjwal Maulik

**Affiliations:** 1 Department of Computer Science and Engineering, University of Kalyani, Kalyani, West Bengal, India; 2 Machine Intelligence Unit, Indian Statistical Institute, Kolkata, West Bengal, India; 3 Department of Computer Science and Engineering, Jadavpur University, Kolkata, West Bengal, India; University of Barcelona, Spain

## Abstract

With the advancement of microarray technology, it is now possible to study the expression profiles of thousands of genes across different experimental conditions or tissue samples simultaneously. Microarray cancer datasets, organized as samples versus genes fashion, are being used for classification of tissue samples into benign and malignant or their subtypes. They are also useful for identifying potential gene markers for each cancer subtype, which helps in successful diagnosis of particular cancer types. In this article, we have presented an unsupervised cancer classification technique based on multiobjective genetic clustering of the tissue samples. In this regard, a real-coded encoding of the cluster centers is used and cluster compactness and separation are simultaneously optimized. The resultant set of near-Pareto-optimal solutions contains a number of non-dominated solutions. A novel approach to combine the clustering information possessed by the non-dominated solutions through Support Vector Machine (SVM) classifier has been proposed. Final clustering is obtained by consensus among the clusterings yielded by different kernel functions. The performance of the proposed multiobjective clustering method has been compared with that of several other microarray clustering algorithms for three publicly available benchmark cancer datasets. Moreover, statistical significance tests have been conducted to establish the statistical superiority of the proposed clustering method. Furthermore, relevant gene markers have been identified using the clustering result produced by the proposed clustering method and demonstrated visually. Biological relationships among the gene markers are also studied based on gene ontology. The results obtained are found to be promising and can possibly have important impact in the area of unsupervised cancer classification as well as gene marker identification for multiple cancer subtypes.

## Introduction

The advent of microarray technology has made it possible the study of the expression profiles of a huge number of genes across different experimental conditions or tissue samples simultaneously. This has significant impact on cancer research. Microarray technology is being utilized in cancer diagnosis through the classification of the tissue samples. When microarray datasets are organized as samples versus gene fashion, then they are very helpful for classification of different types of tissues and identification of those genes whose expression levels are good diagnostic indicators. The microarray datasets, where the tissue samples represent the samples from cancerous (malignant) and non-cancerous (benign) cells, the classification of them will result in binary cancer classification. On the other hand, if the samples are from different subtypes of cancer, then it becomes the problem of multi-class cancer classification. Multi-class cancer classification and detection of gene markers for each cancer subtype is a more challenging task compared to the binary classification.

Most of the researches in the area of cancer diagnosis have focused on supervised classification of cancer datasets through training, validation and testing to classify the tumor samples as malignant or benign, or their subtypes [1]–[6]. However, unsupervised classification or clustering of tissue samples should also be studied since in many cases, labeled tissue samples are not available. In this article, we have explored the application of the multiobjective genetic clustering for unsupervised classification of the tissue samples in multi-class cancer data.

A microarray gene expression dataset consisting of 
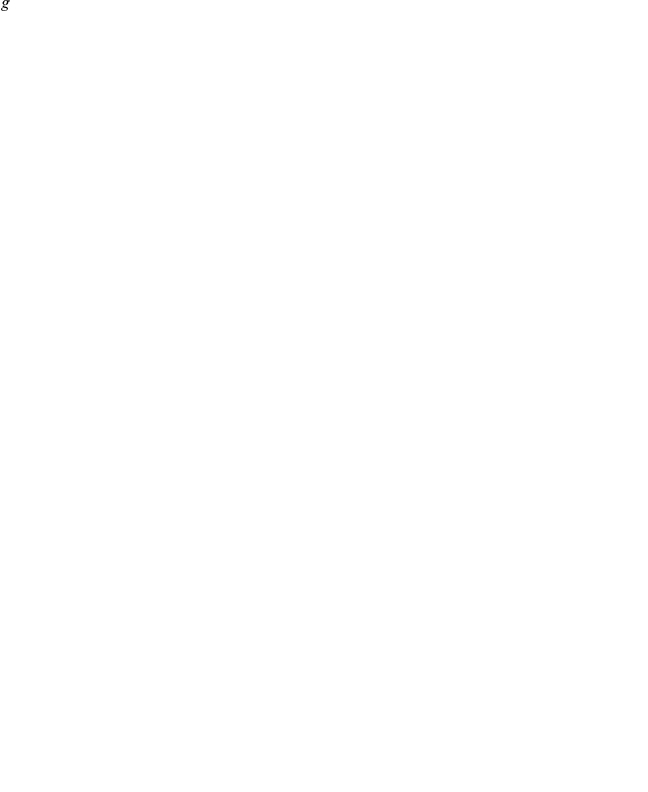
 genes and 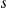 tissue samples is typically organized in a 2D matrix 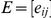 of size 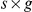. Each element 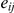 represents the expression level of the 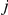
th gene for the 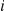
th tissue sample. Clustering [7], [8], an important microarray analysis tool, is used for unsupervised classification of the tissue samples. Clustering methods partition a set of 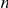 objects into 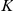 groups based on some similarity/dissimilarity metric where the value of 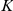 may or may not be known *a priori*.

Genetic algorithms (GAs) [9] have been effectively used to develop efficient clustering techniques [10], [11]. These techniques use a single cluster validity measure as the fitness function to reflect the goodness of an encoded clustering. However, a single cluster validity measure is seldom equally applicable for different data properties. This article poses the problem of clustering as a multiobjective optimization (MOO) [12]–[15] problem. Unlike single objective optimization, in MOO, search is performed over a number of, often conflicting, objective functions. The final solution set contains a number of Pareto-optimal solutions, none of which can be further improved on any one objective without degrading it in another. Non-dominated Sorting Genetic Algorithm-II (NSGA-II) [15], a popular evolutionary multiobjective optimization tool, has been successfully applied in the domain of clustering and classification in microarray gene expression data [16]–[18]. In this article also, an NSGA-II-based multiobjective clustering algorithm [13] has been adopted that optimizes the cluster compactness and cluster separation simultaneously. A challenging issue in MOO is obtaining a final solution from the set of Pareto-optimal solutions. In this regard, a novel method using Support Vector Machine (SVM) [19] classifier is described in this article. The procedure utilizes the points for which most of the non-dominated solutions produce same class labels to train the SVM classifier with a particular kernel. Remaining points are classified by the trained classifier. Final classification is obtained by consensus among the clustering solutions yielded by different kernel functions.

Furthermore, the clustering solution produced by the proposed MOGASVM clustering technique has been used to identify the gene markers that are mostly responsible for distinguishing a particular tumor class from the remaining ones. Signal-to-Noise ratio (SNR) statistic-based gene ranking has been utilized for this purpose.

The performance of the proposed MOGASVM clustering technique has been demonstrated on three publicly available benchmark cancer datasets, viz., SRBCT, Adult malignancy and Brain tumor. The superiority of the proposed technique, as compared to K-means clustering [7], Expectation Maximization (EM) clustering [20], single objective GA-based clustering that optimizes the combination of cluster compactness and separation (SGA), hierarchical average linkage clustering [7], Self Organizing Map (SOM) clustering [21], consensus clustering [22] and a recently proposed clustering technique called SiMM-TS [12], is demonstrated both quantitatively and visually. The superiority of the MOGASVM clustering technique has also been proved to be statistically significant through statistical significance tests. Finally, it has been demonstrated how the MOGASVM clustering result can be used for identifying the relevant gene markers for the SRBCT datasets. Also a study of biological relevance of the gene markers have been conducted based on gene ontology.

## Materials and Methods

### Multiobjective Optimization using Genetic Algorithms

In many real world situations there may be several objectives that must be optimized simultaneously in order to solve a certain problem. This is in contrast to the problems tackled by conventional GAs, which involve optimization of just a single criterion. The main difficulty in considering multiobjective optimization is that there is no accepted definition of optimum in this case, and therefore it is difficult to compare one solution with another. In general, these problems admit multiple solutions, each of which is considered acceptable and equivalent when the relative importance of the objectives is unknown. The best solution is subjective and depends on the need of the designer or decision maker.

Traditional search and optimization methods such as gradient descent search, and other unconventional ones such as simulated annealing are difficult to extend as it is to the multiobjective case, since their basic design precludes the consideration of multiple solutions. On the contrary, population based methods like evolutionary algorithms are well suited for handling such situations. The multiobjective optimization can be formally stated as [23], [24]. Find the vector 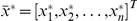 of decision variables which satisfies 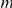 inequality constraints:
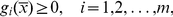
(1)

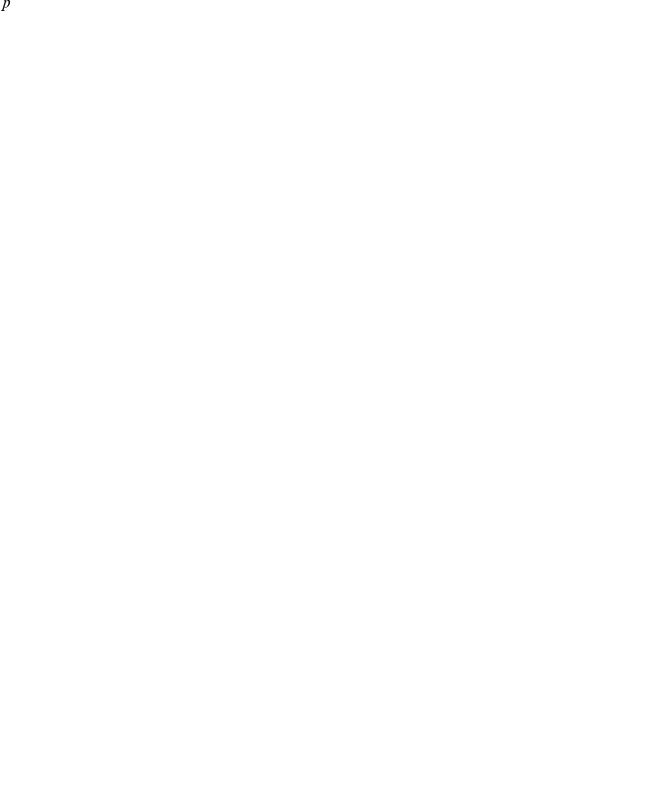
 equality constraints
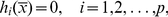
(2)and optimizes the vector function
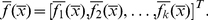
(3)The constraints given in Eqns. (1) and (2) define the feasible region 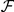 which contains all the admissible solutions. Any solution outside this region is inadmissible since it violates one or more constraints. The vector 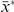 denotes an optimal solution in 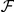. In the context of multiobjective optimization, the difficulty lies in the definition of optimality, since it is only rarely that we will find a situation where a single vector 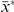 represents the optimum solution to all the objective functions.

The concept of *Pareto-optimality* is useful in the domain of multiobjective optimization. A formal definition of Pareto-optimality from the viewpoint of minimization problem may be given as follows. A decision vector 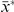 is called Pareto-optimal if and only if there is no 
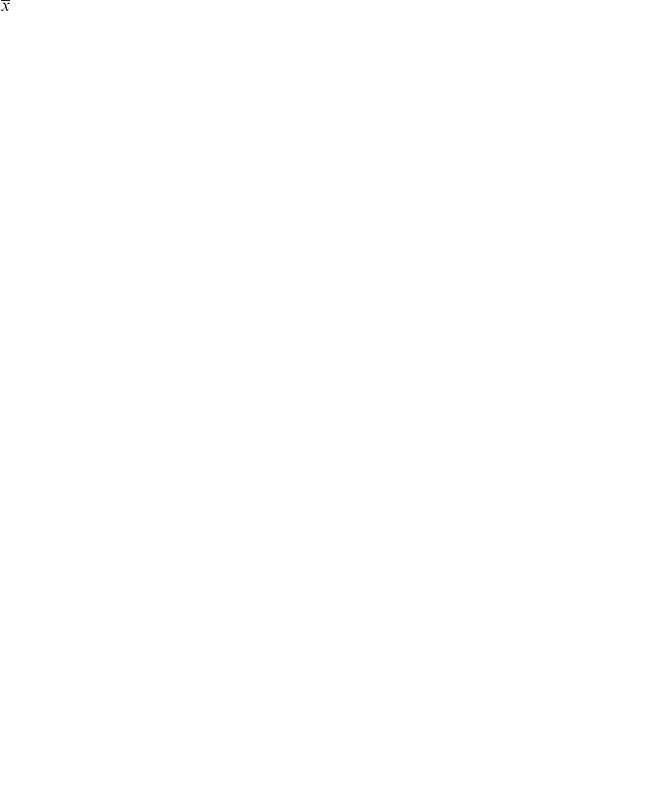
 that dominates 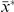, i.e., there is no 
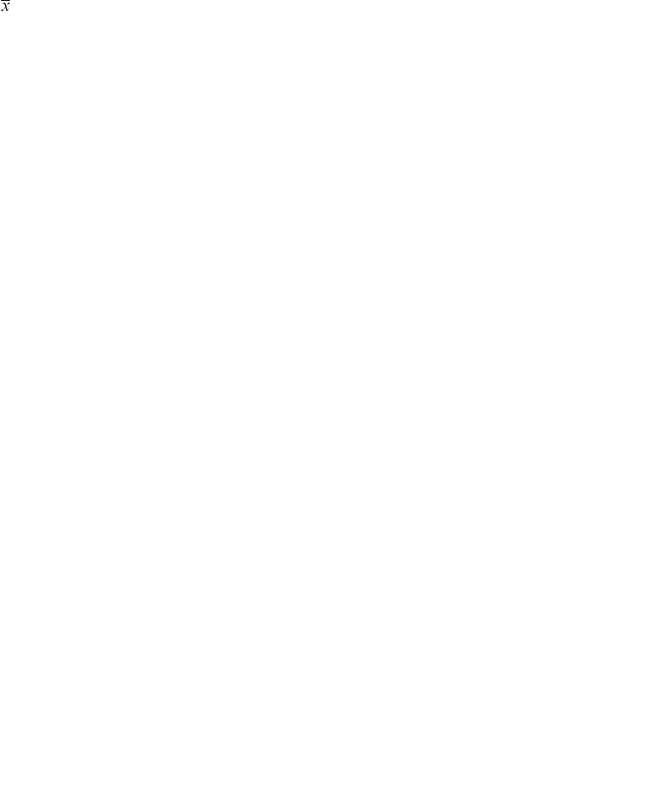
 such that
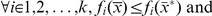


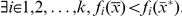
In other words, 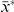 is Pareto-optimal if there exists no feasible vector 
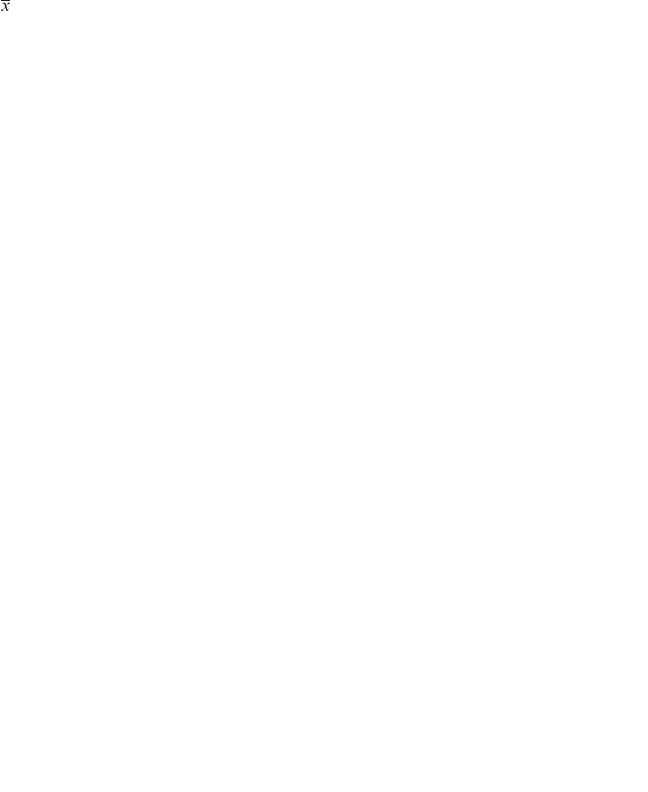
 which causes a reduction on some criterion without a simultaneous increase in at least another. In this context, two other notions viz., *weakly non-dominated* and *strongly non-dominated* solutions are defined [23]. A point 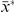 is a weakly non-dominated solution if there exists no 
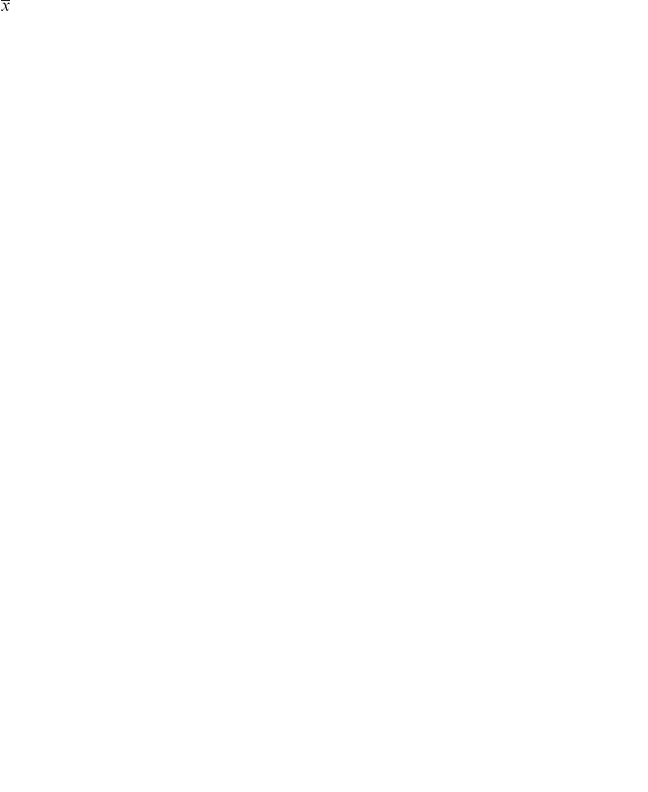
 such that 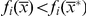, for 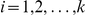. A point 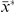 is a strongly non-dominated solution if there exists no 
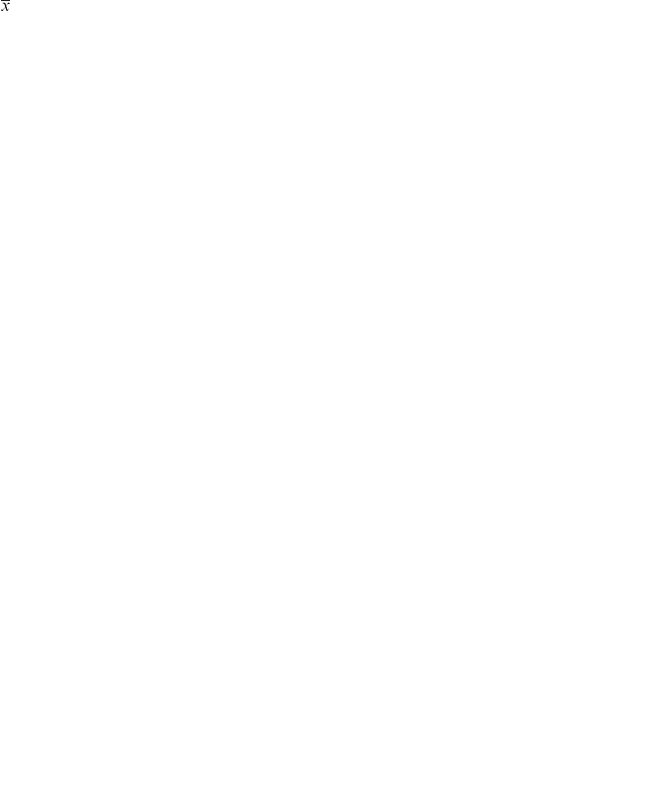
 such that 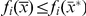, for 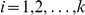, and for at least one 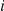, 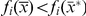. In general, Pareto optimum admits a set of solutions called *non-dominated* solutions.

There are different approaches for solving multiobjective optimization problems [23], [24], e.g., aggregating, population based non-Pareto and Pareto-based techniques. In aggregating techniques, the different objectives are generally combined into one using weighting or goal based method. Vector Evaluated Genetic Algorithm (VEGA) is a technique in the population based non-Pareto approach in which different subpopulations are used for the different objectives. Multiple Objective GA (MOGA), Non-dominated Sorting GA (NSGA), Niched Pareto GA (NPGA) constitute a number of techniques under the Pareto-based approaches. However, all these techniques, described in [24], are essentially non-elitist in nature. NSGA-II [15], Strength Pareto Evolutionary Algorithm (SPEA) [25] and SPEA2 [26] are some more recent elitist techniques. NSGA-II is an improvement over its previous version NSGA in terms computation time. Moreover, NSGA-II introduces a novel elitist model by combining the parent and child populations and propagating the non-dominated solutions from the combined population to the next generation ensuring better convergence rate towards globally optimal Pareto front. Also it proposes a crowded comparison method for binary tournament selection that provides better diversity in the Pareto front. In [15], it has been shown that NSGA-II performs better compared to several other MOO techniques. Hence the multiobjective clustering technique considered in this work uses NSGA-II as the underlying optimization framework. However, any other evolutionary multiobjective optimization tool could have been used.

### NSGA-II based Multiobjective Clustering

In this section, we have described the use of NSGA-II for evolving a set of near-Pareto-optimal clustering solutions [13]. Cluster compactness and the cluster separation are considered as the objective functions that are optimized simultaneously. The technique is described below in detail.

#### String Representation and Population Initialization

In the NSGA-II based clustering, the chromosomes are made up of real numbers which represent the coordinates of the centers of the clusters. Suppose the size of the dataset is 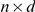, i.e., the algorithm clusters 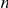 tissue samples each of which is described by 
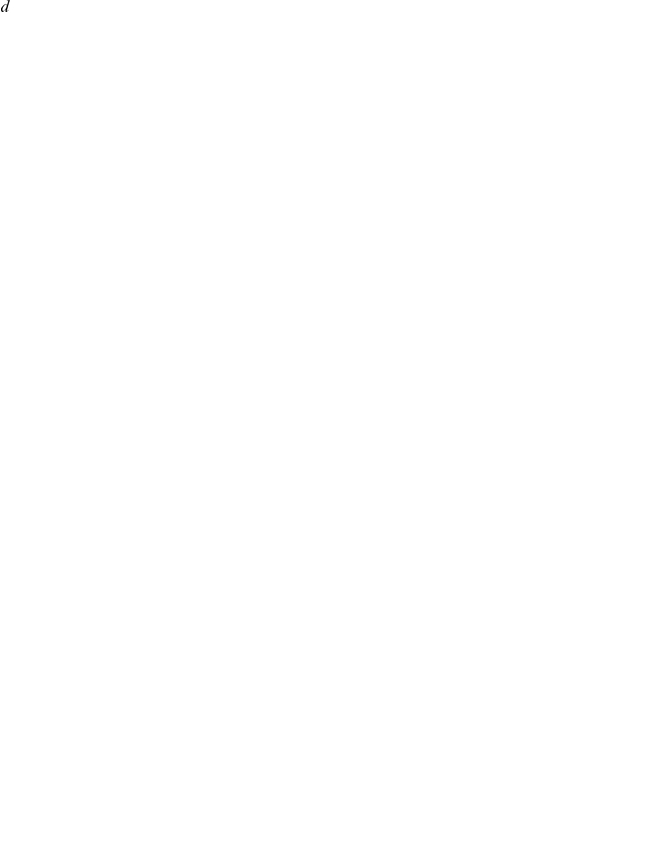
 genes (features). For 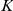 clusters, each chromosome thus has a length of 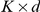, where 
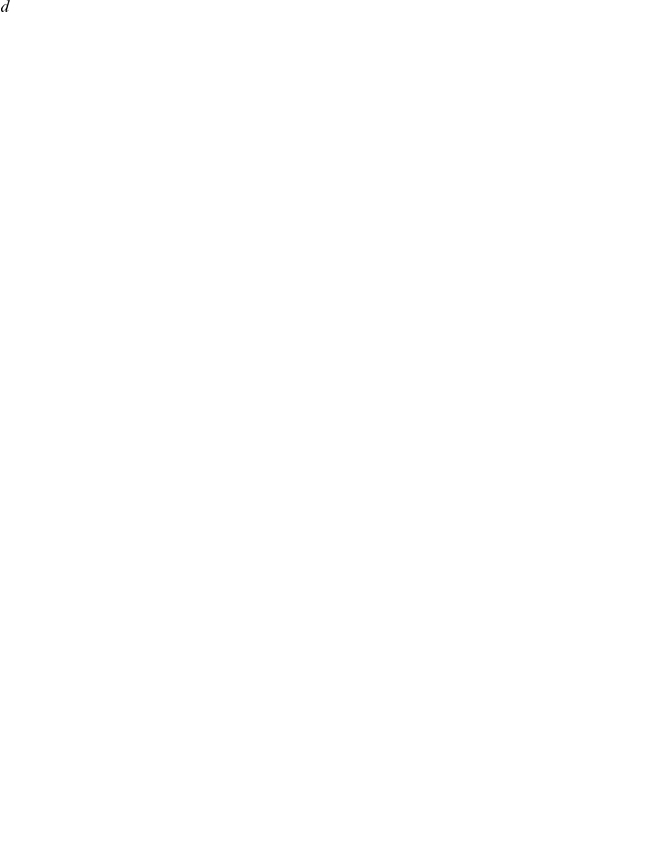
 is the data dimension (the number of genes in this case). As we have used 200 genes that have larger variances across the samples, the dimension 
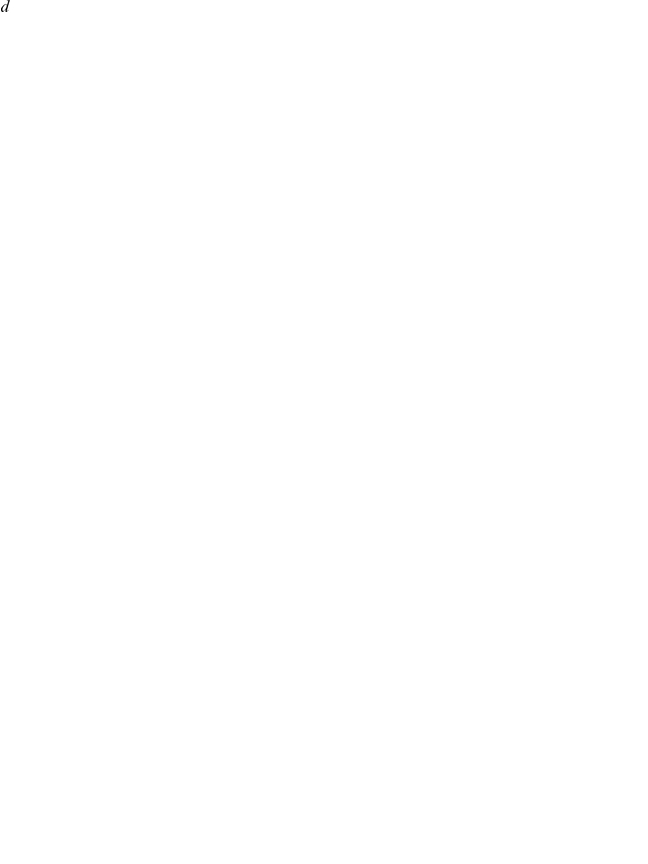
 is therefore 200 for each dataset. The centers encoded in a chromosome in the initial population are randomly selected 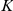 distinct points from the dataset.

#### Computing the Objectives

For computing the objective functions, first the centers 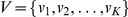 encoded in a given chromosome are extracted. Thereafter, each data point is assigned to its nearest cluster center and the cluster centers are updated by taking the mean of the points assigned to it. The points are then reassigned to their nearest cluster centers. The chromosome is also updated with the new cluster centers.

The global compactness 
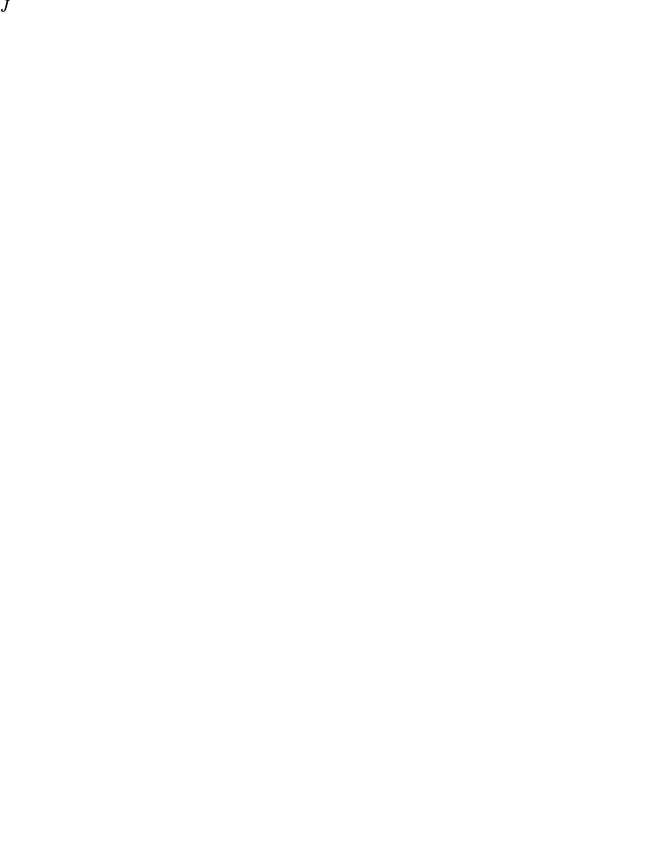
 of a clustering solution is defined as follows:
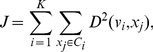
(4)where 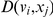 denotes the distance between the 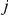
th point and 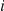
th cluster center. 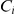 denotes the 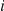
th cluster. Note that low value of 
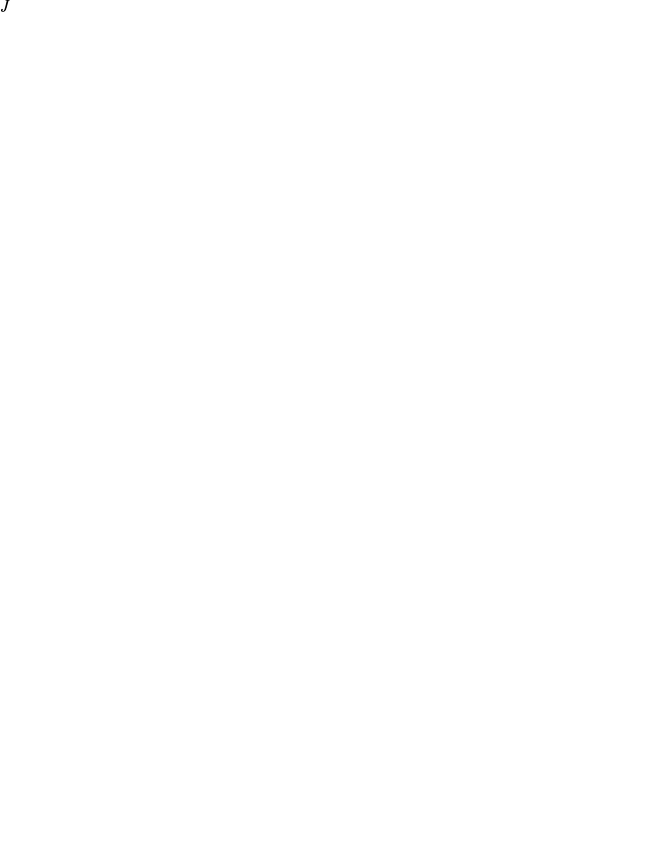
 indicates that the clusters are highly compact. Hence the objective is to minimize 
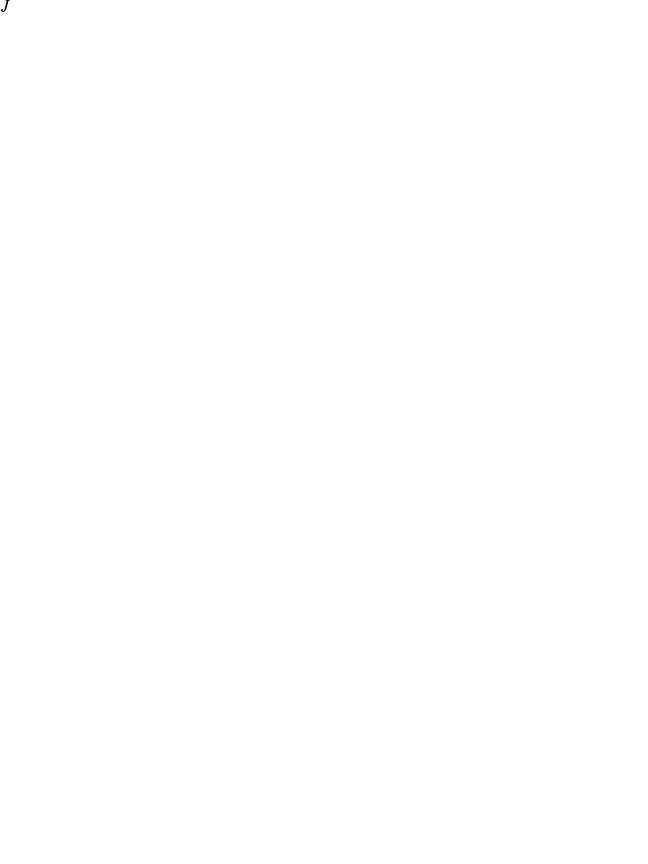
.

The second objective is cluster separation 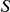. This is defined as follows:
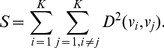
(5)To obtain well separated clusters, the objective 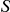 is to be maximized. As here NSGA-II is modeled as a minimization problem, the second objective is taken as the reciprocal of 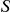.

#### Genetic Operations

The popularly used genetic operations are *selection*, *crossover* and *mutation*. The selection operation used here is the crowded binary tournament selection used in NSGA-II [15]. After selection, the selected chromosomes are put in the mating pool and conventional single point crossover is performed based on the crossover probability 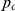. After that, each chromosome undergoes mutation depending on the mutation probability 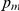, where a random cluster center is chosen from it and then moved slightly.

The most characteristic part of NSGA-II is its elitism operation, where the parent and child populations are combined and the non-dominated solutions from the combined population are propagated to the next generation. For the details on the different genetic processes, the readers may refer to [15]. The near-Pareto-optimal strings of the last generation provide the different solutions to the clustering problem.

### Support Vector Machine Classifier

Support vector machine (SVM) classifiers are inspired by statistical learning theory and they perform structural risk minimization on a nested set structure of separating hyperplanes [19], [27]. Viewing the input data as two sets of vectors in a 
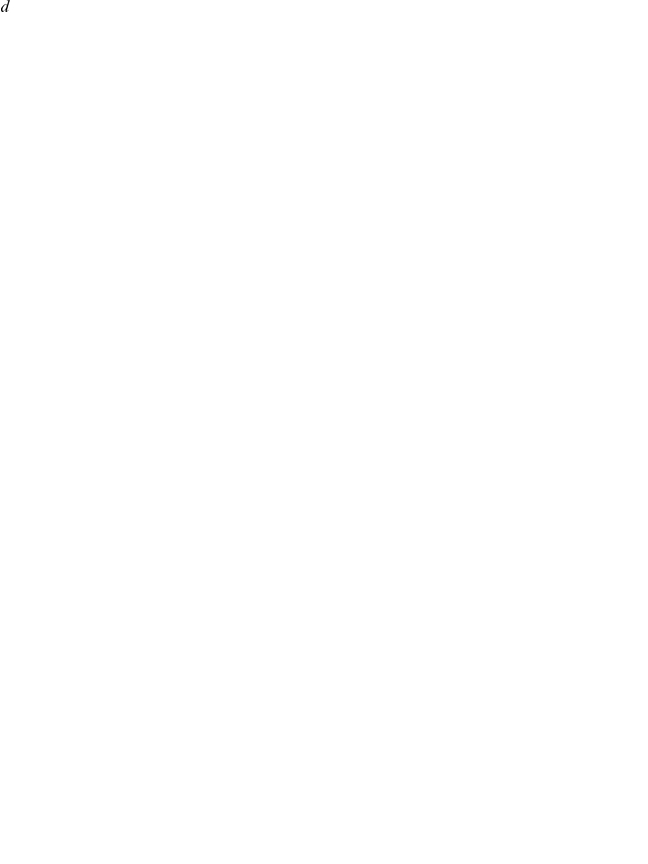
-dimensional space, an SVM constructs a separating hyperplane in that space, which maximizes the margin between the two classes of points. To compute the margin, two parallel hyperplanes are constructed on each side of the separating one, which are “pushed up against” the two classes of points. Intuitively, a good separation is achieved by the hyperplane that has the largest distance to the neighboring data points of both classes. Larger margin or distance between these parallel hyperplanes indicates better generalization error of the classifier. Fundamentally, the SVM classifier is designed for two-class problems. It can be extended to handle multi-class problems by designing a number of one-against-all or one-against-one two-class SVMs.

Suppose a dataset consists of 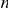 feature vectors 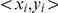, 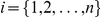, where 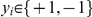, denotes the class label for the data point 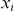. The problem of finding the weight vector 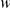 can be formulated as minimizing the following function:
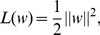
(6)subject to
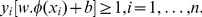
(7)Here, 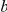 is the bias and the function 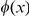 maps the input vector to the feature vector. The dual formulation is given by maximizing the following:
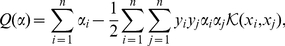
(8)subject to
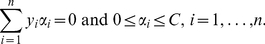
(9)Only a small fraction of the 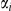 coefficients are nonzero. The corresponding pairs of 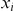 entries are known as support vectors and they fully define the decision function. Geometrically, the support vectors are the points lying near the separating hyperplane. Here 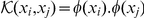 is called the *kernel function*.

Kernel functions help to map the feature space into higher dimensional space. The kernel function may be linear or non-linear, like polynomial, sigmoidal, radial basis functions (RBF), etc. The four kernel functions used in this article are as follows:

Linear: 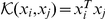


Polynomial: 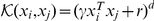


Sigmoidal: 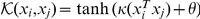


Radial Basis Function (RBF): 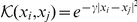.

The extended version of the two-class SVM that deals with multi-class classification problem by designing a number of one-against-all two-class SVMs [27] is used here. For example, a 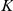-class problem is handled with 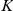 two-class SVMs, each of which is used to separate a class of points from all the remaining points.

### Obtaining the Final Clustering from the Non-dominated Solutions

As the multiobjective clustering produces a set of non-dominated solutions in the final generation, it is required to apply some technique to obtain the final clustering solution from this set. This section describes the proposed scheme for combining the NSGA-II-based multiobjective clustering algorithm with the SVM classifier for this purpose. In the combined approach, named MOGASVM, each non-dominated solution is given equal importance and a majority voting technique is applied. This is motivated by the fact that due to the presence of training points, supervised classification usually performs better than the unsupervised classification or clustering. Here we have exploited this advantage while selecting some training points using majority voting on the non-dominated solutions produced by the multiobjective clustering. The majority voting technique gives a set of points for which most of the non-dominated solutions assign the same class labels. Hence these points can be thought to be clustered properly and thus can be used as the training points of the SVM classifier. Subsequently, the remaining low-confidence points are classified using the trained classifier. The process is repeated for different kernel functions and the final clustering is obtained through majority voting among the cluster label vectors produced by the different kernel functions. The steps of MOGASVM are described below.

**Step 1:** Execute MOGA clustering to obtain a set 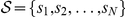, 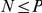, of non-dominated solution strings consisting of cluster centers.

**Step 2:** Decode each solution 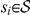 and obtain the cluster label vector for each solution by assigning each point to its nearest cluster center.

**Step 3:** Reorganize the cluster label vectors to make them consistent, i.e., cluster 
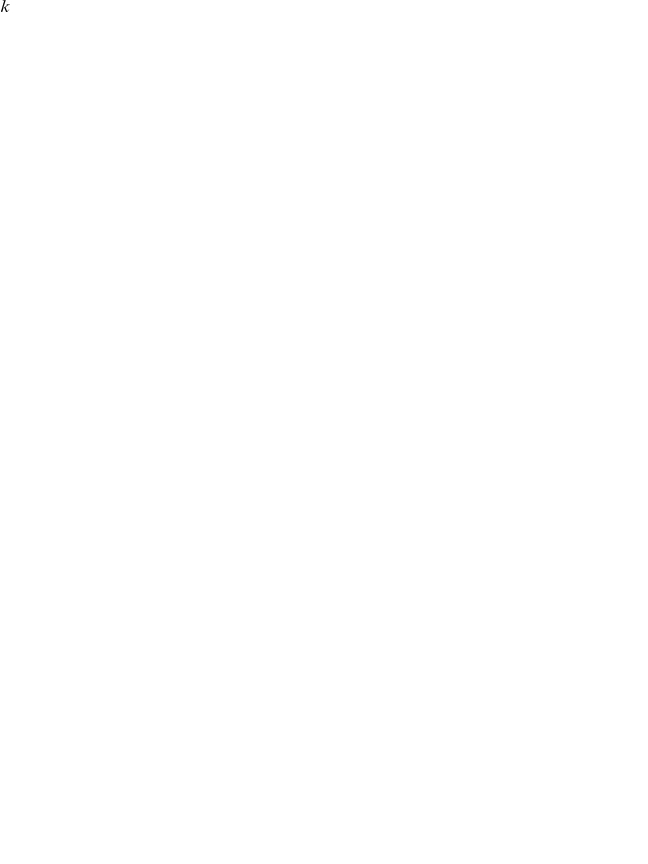
 in the first solution should correspond to cluster 
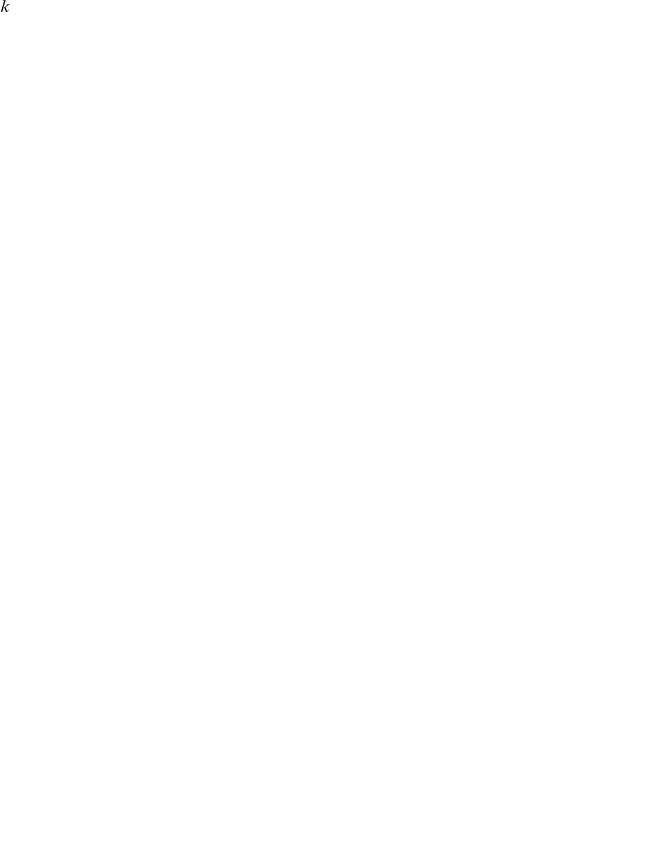
 in all other solutions. For example, the cluster label vector 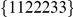 is equivalent to 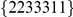.

**Step 4:** Mark the points which are given the same class label 
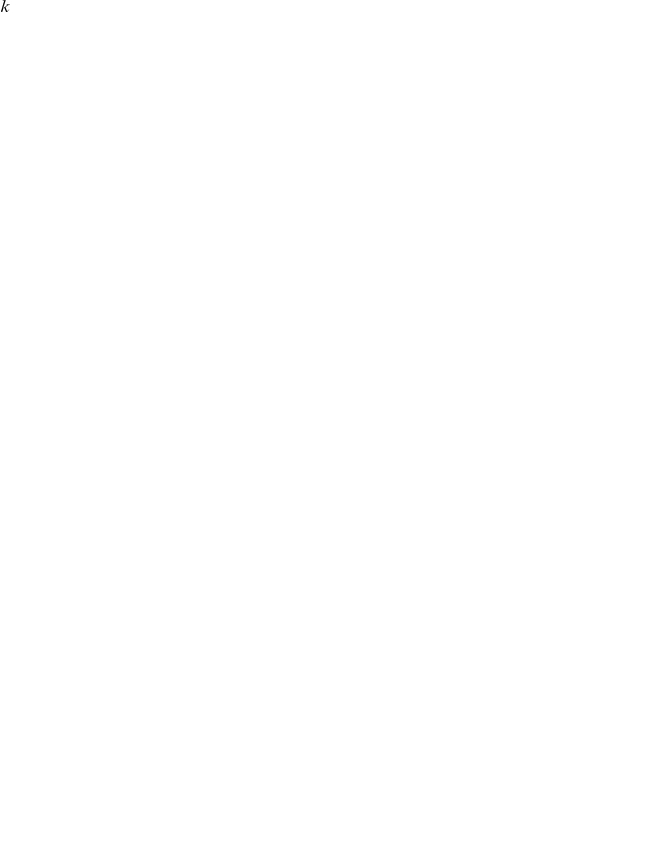
 for at least 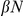 solutions, as the training points, where 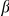, 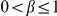, is the majority voting threshold. The class labels of the points will be class 
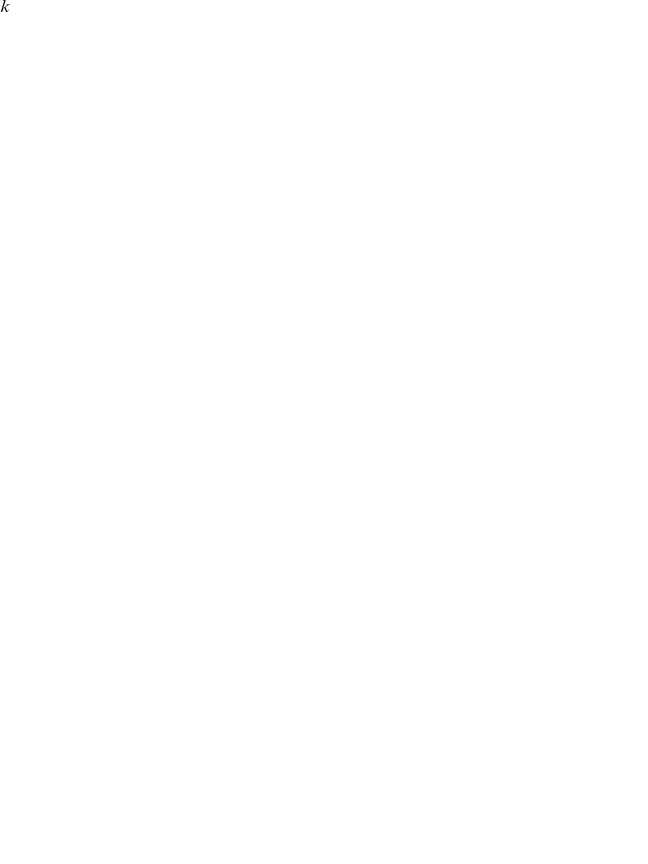
.

**Step 5:** Train the SVM classifier with some kernel function using the training points.

**Step 6:** Generate the class labels for the remaining points using the trained SVM classifier.

**Step 7:** Repeat Steps 5–6 for the four kernel functions considered here and obtain the four cluster label vectors.

**Step 8:** Combine the four clustering label vectors through majority voting ensemble, i.e., each point is assigned a class label that obtains the maximum number of votes among the four clustering solutions. Ties are broken randomly.

The sizes of the training and testing sets depend on the parameter 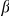 (majority voting threshold), which determines the minimum number of non-dominated solutions that must agree with each other in the voting context. If 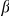 has a high value, the size of the training set is small. However it implies that more number of non-dominated solutions agree with each other and thus confidence of the training set is high. On the contrary, if 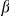 has a low value, the size of the training set is large. But it indicates that less number of non-dominated solutions have agreement among themselves and the training set has low confidence level. During experimentation, we have tried different values for 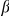 and found that the performance of MOGASVM is in general best when 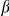 is in the range between 0.4 and 0.6. This has been observed for all the datasets considered here. Therefore, to achieve a tradeoff between the size and confidence of the training set, after several experiments, we have set the parameter 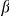 to a value of 0.5. However, this parameter can be exposed to the user who can tune it according to his/her need.

### Number of Clusters

For setting the number of clusters 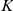, silhouette index is used [28]. It is defined as follows. Suppose 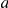 represents the average distance of a point from the other points of the cluster to which the point is assigned, and 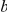 represents the minimum of the average distances of the point from the points of the other clusters. Now the silhouette width 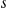 of the point is defined as:
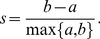
(10)Silhouette index 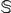 is the average silhouette width of all the data points (tumor samples) and it reflects the compactness and separation of the clusters. The value of silhouette index varies from −1 to 1 and higher value indicates better clustering result. The value of 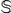 does not have any monotonic increasing or decreasing tendency with the number of clusters. Hence this index is a good indicator for selecting the number of clusters [28].

To select the number of clusters 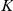, the MOGASVM algorithm is run for different values of 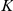 starting from 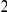 to 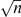, 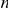 being the number of data points. For each 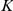, it is executed 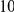 times from different initial configurations and the run giving the best 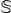 value is taken. Among these best solutions for different 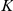 values, the value of 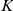 for the solution producing the maximum 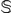 index value is chosen. The same 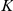 value is used for all the algorithms for a fair comparison.

### Dealing with the Outliers

It is known that the presence of outliers can affect the performance of the clustering algorithms. The proposed MOGASVM clustering algorithm computes the means of the clusters during chromosome updation which is likely to be affected due to the presence of outliers in the dataset. To cope with this, we modified the proposed algorithm as follows. During the chromosome updation, instead of taking the means of the points in a cluster, we compute the *medoid* of the cluster. A cluster medoid, unlike cluster mean, is an actual data point in the cluster from which the sum of the distances to the other points of the cluster is minimum. Since medoid is an actual data point, it is less influenced by the presence of outliers [29]. The rest of the steps of the modified algorithm remains same. During experimentation, it has been found that the medoid-based multiobjective clustering algorithm performs similarly as the mean-based approach for the three datasets considered in this article. Therefore we have not reported the results for the medoid-based approach. This suggests that the datasets considered here are possibly free from outliers. However, this may not be true for the other datasets and in that case, it will be better to use the medoid-based approach instead of the mean-based one. It is to be noted that finding the medoids is computationally more expensive than finding the means. But it is possible to precompute the complete distance matrix and keep it in memory during the execution of the clustering algorithm for faster performance, because the number of samples in sample-gene microarray datasets is usually much smaller compared to the number of genes.

### Performance Metrics

Two performance measures, i.e., percentage Classification Accuracy (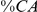) and Adjusted Rand Index (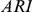) are considered for comparing the results produced by different algorithms. These are defined below.

#### Percentage Classification Accuracy

We define the percentage Classification Accuracy (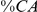) to compare a clustering solution with the true clustering. Suppose 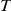 is the true clustering of the samples in a gene expression dataset and 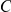 is a clustering result given by some clustering algorithm. Let 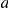 be the number of pairs of points that belong to the same clusters in both 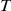 and 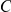, 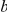 be the number of pairs of points that belong to different clusters in both 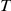 and 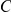, and 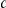 be the total number of pairs of points, i.e., 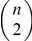. The 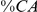 is defined as:
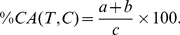
(11)Higher value of 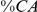 means a better matching between 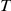 and 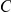. Evidently 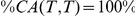.

#### Adjusted Rand Index

The Adjusted Rand index (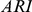) [30] is also used to compare a clustering solution with the true clustering. Suppose 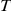 is the true clustering of the samples in a gene expression dataset and 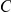 is a clustering result given by some clustering algorithm. Let 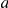, 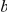, 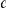 and 
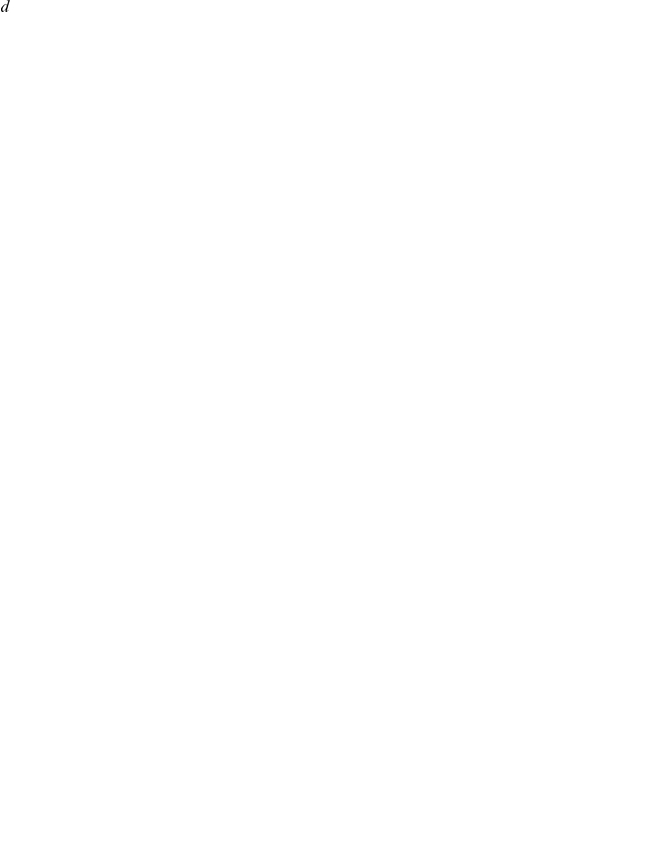
 respectively denote the number of pairs of points belonging to the same cluster in both 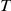 and 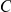, the number of pairs belonging to the same cluster in 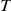 but to different clusters in 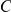, the number of pairs belonging to different clusters in 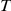 but to the same cluster in 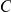, and the number of pairs belonging to different clusters in both 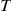 and 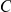. The adjusted Rand index 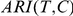 is then defined as follows:
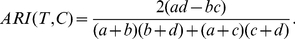
(12)The value of 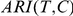 lies between 0 and 1 and higher value indicates that 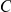 is more similar to 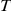. Evidently, 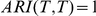.

### Identification of the Gene Markers

In this section we have demonstrated how the proposed MOGASVM clustering technique can be used to identify the gene markers that are mostly responsible for distinguishing the different classes of tissue samples. Here we have demonstrated the process for the SRBCT dataset (described in the next section). This has been done as follows.

At first, MOGASVM is applied to cluster the samples of the preprocessed dataset into four classes corresponding to the tumor subtypes EWS, NB, BL and RMS, respectively. To obtain the gene markers for the EWS subtype, the clustering result is treated as two classes: one class corresponds to the EWS tumors and the other class corresponds to the remaining tumor types. Considering these two classes, for each of the genes, a statistic called Signal-to-Noise Ratio (SNR) [1] is computed. The SNR is defined as
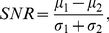
(13)where 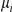 and 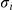, 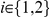, respectively denote the mean and standard deviation of class 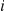 for the corresponding gene. Note that larger absolute value of SNR for a gene indicates that the gene's expression level is high in one class and low in another. Hence this bias is very useful in distinguishing the genes that are expressed differently in the two classes of samples. After computing the SNR statistic for each gene, the genes are sorted in descending order of their SNR values. From the sorted list, top 10 genes are selected as the gene markers (5 down-regulated, i.e., negative SNR and 5 up-regulated, i.e., positive SNR) for the EWS subtype. The top 10 gene markers for the other tumor subtypes are selected similarly, i.e., by considering two classes each time, one corresponding to the tumor class for which the gene markers are being identified, and the other corresponding to all the remaining tumor classes.

It has been observed that the set of top 10 genes selected in different runs of MOGASVM varies slightly from one run to another. So while reporting the final gene markers for the SRBCT data, we have reported the most frequently selected 10 genes over all the runs. The frequencies of the selected genes have also been reported. Moreover, the clustering result obtained using the 40 marker genes for the SRBCT data (10 for each of the 4 cancer subtypes) is compared with the clustering results obtained using initially selected 200 genes to show the effectiveness of using only the marker genes for clustering.

### Datasets

In this article, three publicly available benchmark cancer datasets, viz., *SRBCT*, *Adult malignancy* and *Brain tumor* datasets have been used for experiments. The datasets are described in this section.

#### Small Round Blood Cell Tumors (SRBCT)

The small round blood cell tumors (SRBCT) are 4 different childhood tumors named so because of their similar appearance on routine histology [5]. The number of samples is 63 and total number of genes is 2308. They include Ewing's family of tumors (EWS) (23 samples), neuroblastoma (NB) (8 samples), Burkitt's lymphoma (BL) (12 samples) and rhabdomyosarcoma (RMS) (20 samples). This dataset is publicly available at http://www.ailab.si/supp/bi-cancer/projections/info/SRBCT.htm.

#### Adult Malignancy

This data consists of 190 tumor samples, spanning 14 common tumor types to oligonucleotide microarray [6]. The 14 tumor types are: breast adenocarcinoma (BR) (11 samples), prostate adenocarcinoma (PR) (10 samples), lung adenocarcinoma (LU) (11 samples), colorectal adenocarcinoma (CR) (11 samples), lymphoma (LY) (22 samples), bladder transitional cell carcinoma (BL) (10 samples), melanoma (ML) (11 samples), uterine adenocarcinoma (UT) (10 samples), leukemia (LE) (30 samples), renal cell carcinoma (RE) (11 samples), pancreatic adenocarcinoma (PA) (11 samples), ovarian adenocarcinoma (OV) (11 samples), pleural mesothelioma (ME) (11 samples) and central nervous system (CNS) (20 samples). The number of genes is 1363. This dataset is publicly available at the following website: http://algorithmics.molgen.mpg.de/Static/Supplements/CompCancer.

#### Brain Tumor

Embryonal tumors of the central nervous system (CNS) represent a heterogeneous group of tumors [6]. The dataset contains five types of tumor samples viz., primitive neuroectodermal tumors (PNETs) (8 samples), atypical teratoid/rhabdoid tumors (Rhab) (10 samples), malignant gliomas (Mglio) (10 samples), medulloblastomas (MD) (10 samples) and normal tissues (Ncer) (4 samples). The number of genes in this dataset is 1379. This dataset is also publicly available at the following website: http://algorithmics.molgen.mpg.de/Static/Supplements/CompCancer.

### Preprocessing of the Datasets

Each dataset is subjected to the following preprocessing steps to find out the genes with most variability across the samples. At first, for each gene, we have calculated its variance across all the samples. Thereafter, the genes are sorted in descending order of their variances. Subsequently, from all the genes, the top 200 genes with the largest variance across the samples are selected. This is done with the expectation that the genes having larger variance across the samples are more effective in distinguishing different classes of tumor samples rather than the genes with small variance across the samples. Next, the expression values are log-transformed. Finally, each sample is normalized to have mean 0 and variance 1.

## Results and Discussion

The performance of the proposed MOGASVM clustering has been compared with that of K-means clustering [20], Expectation Maximization (EM) clustering [20], single objective GA minimizing 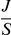 (SGA), hierarchical average linkage clustering [7], Self Organizing Map (SOM) clustering [21], SiMM-TS clustering [12] and consensus clustering [22]. Under consensus clustering, three cluster ensemble approaches as found in [22], namely, Cluster-based Similarity Partitioning Algorithm (CSPA), HyperGraph Partitioning Algorithm (HGPA) and Meta-CLustering Algorithm (MCLA) are studied. The clustering solutions found by the K-means, EM, average linkage and SOM clustering have been combined through these three cluster ensemble techniques. We have tested two well-known distance measures viz., Euclidean and Pearson Correlation based distance. However as the datasets are normalized so that each row has mean 0 and variance 1, it is known that both Euclidean and correlation based distance perform similarly. Therefore, in this section, we have reported the results for Euclidean distance only.

### Input Parameters

The different parameters of MOGA and SGA are taken as follows: number of generations = 100, population size = 50, crossover probability = 0.8 and mutation probability = 0.01. The value of the parameters 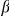 is taken as 0.5. The parameters have been set after several experiments. It has been found during experimentation that the best clustering is actually obtained with lower number of generations and smaller population size for all the datasets. However, to make it standard and consistent for all the datasets considered here, we have chosen the aforementioned parameter setting to obtain good clustering result within reasonable time. The probabilities of crossover and mutation are also selected experimentally and found to be reasonably robust around the selected values. The K-means, EM and SOM clustering have been run for 5000 iterations unless they converge before that. In each run of hierarchical average linkage clustering, K-means, EM and SOM, the clustering solutions are combined using CSPA, HGPA and MCLA ensemble approaches to obtain the consensus clustering.

### Clustering Performance

Firstly, in Table 1, we have reported the average 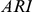 index and 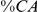 index scores over 50 consecutive runs of MOGASVM (with majority voting ensemble of four kernel functions) and MOGASVM with individual kernel functions for the three datasets considered in this article. It is evident from the performance index scores that the ensemble of kernel functions performs better than the individual kernel functions. This demonstrates the utility of MOGASVM with ensemble of kernel functions rather than using the four kernel functions individually.

**Table 1:** The comparison of the average ARI and %CA scores produced by 50 consecutive runs of MOGASVM with ensemble of kernel functions and MOGASVM with individual kernel functions for all the datasets.

Algorithms	SRBCT		Adult malignancy		Brain tumor	
	ARI	%CA	ARI	%CA	ARI	%CA
MOGASVM	0.5126	76.6412	0.8172	96.4718	0.7172	88.5150
MOGASVM (linear)	0.4726	74.7926	0.7591	95.8244	0.6836	87.5836
MOGASVM (polynomial)	0.4682	74.5343	0.7238	94.7375	0.6927	88.0116
MOGASVM (sigmoidal)	0.4816	76.0284	0.7704	95.7581	0.6734	87.2046
MOGASVM (RBF)	0.4855	76.2891	0.7926	96.2183	0.7025	88.1173

Tables 2,3,4 report the average 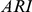 index and average 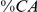 index scores over the 50 runs of each algorithm considered in this article, respectively, for the SRBCT, Adult malignancy and Brain tumor datasets. For all the three datasets, the silhouette index has found the correct number of clusters. As is evident from the tables, MOGASVM produces the best average 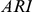 index and 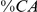 index scores compared to the other algorithms.

**Table 2:** The average 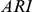 and 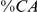 scores produced by 50 consecutive runs of different algorithms for the SRBCT data.

Algorithms	ARI	%CA
MOGASVM	0.5126	76.6412
K-means	0.3135	70.1903
EM	0.3376	71.1295
SGA	0.3198	70.8193
Avg. linkage	0.1021	49.0527
SOM	0.3872	71.7845
SiMM-TS	0.4628	74.4853
CSPA	0.3922	72.0297
HGPA	0.2839	67.4533
MCLA	0.3902	71.9764

**Table 3:** The average 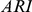 and 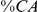 scores produced by 50 consecutive runs of different algorithms for the Adult malignancy data.

Algorithms	ARI	%CA
MOGASVM	0.8172	96.4718
K-means	0.6924	92.5441
EM	0.7251	94.7294
SGA	0.7491	95.7858
Avg. linkage	0.6190	93.0437
SOM	0.5917	92.8100
SiMM-TS	0.7823	96.0139
CSPA	0.7331	95.0801
HGPA	0.7192	94.0549
MCLA	0.7398	95.2813

**Table 4:** The average 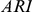 and 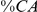 scores produced by 50 consecutive runs of different algorithms for the Brain tumor data.

Algorithms	ARI	%CA
MOGASVM	0.7172	88.5150
K-means	0.5764	84.5144
EM	0.5581	83.1457
SGA	0.6325	87.1433
Avg. linkage	0.4603	78.2811
SOM	0.6214	87.0376
SiMM-TS	0.6892	87.9110
CSPA	0.6028	85.9984
HGPA	0.5295	83.9416
MCLA	0.5974	86.4543

From the tables, it appears that MOGASVM also outperforms its single objective counterpart SGA that optimizes the combination of cluster compactness and cluster separation. On the other hand, MOGASVM optimizes both of these objectives simultaneously. As MOGASVM performs better in terms of both the performance scores, it indicates that optimizing multiple criteria simultaneously can yield better clustering rather than the case when the objectives are combined into one.

**Figure 1:**
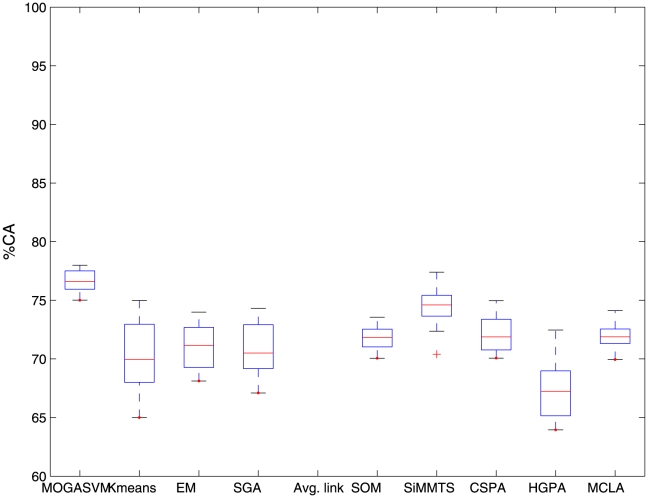
The boxplots showing the 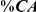 index scores produced by different algorithm over 50 consecutive runs for the SRBCT dataset.

**Figure 2:**
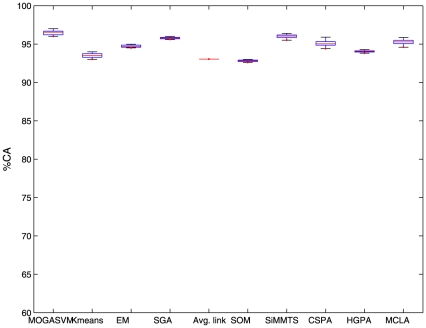
The boxplots showing the 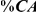 index scores produced by different algorithm over 50 consecutive runs for the Adult malignancy dataset.

**Figure 3:**
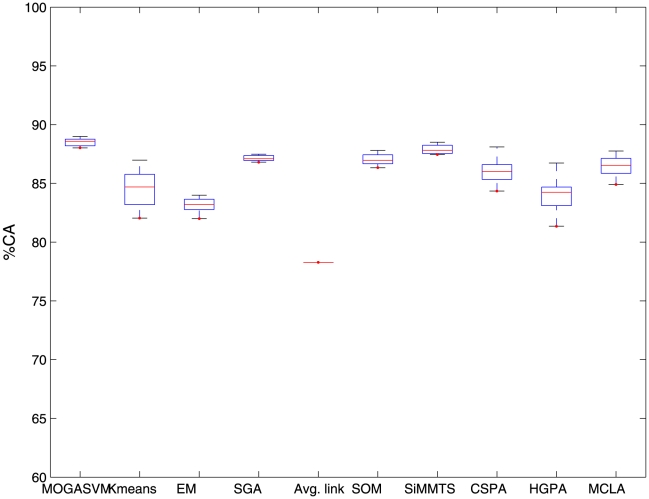
The boxplots showing the 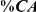 index scores produced by different algorithm over 50 consecutive runs for the Brain tumor dataset.

For the purpose of illustration, Figures 1,2,3 show the boxplots representing the 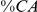 scores over 50 runs of the algorithms for the three datasets considered here. It is evident from the figures that the boxplots corresponding to MOGASVM are situated at the upper side of the figures, which indicates that MOGASVM produces higher 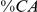 scores than those produced by the other algorithms. SiMM-TS has been found to be the closest competitor of MOGASVM for all the datasets.

### Execution Time

All the algorithms have been implemented in Matlab and executed on an Intel Core 2 Duo 2.0 GHz Machine with 2 GB memory having Windows XP operating system. It should be noted that time requirement for the GA based clustering techniques is usually more because of the different genetic operations performed during the execution of the algorithms. On average, MOGASVM executes for 97.24 seconds for the SRBCT dataset, whereas the SGA and SiMM-TS clustering takes 75.93 and 161.37 seconds, respectively. The other algorithms execute only for a few seconds for this dataset. The execution times have been computed on the basis of the parameter settings discussed in the Input Parameters section. As expected, the execution time of MOGASVM is larger compared to the other single objective clustering methods because of some additional operations necessitated by its multiobjective nature. Only SiMM-TS takes more time than it, because SiMM-TS needs two stages of clustering. The iterative algorithms have always converged far before reaching the maximum number of iterations. However, as is evident from the results, the clustering performance of MOGASVM is the best among all the methods for all the datasets considered in this article. It is also found during experimentation that even if the other algorithms used for comparison are allowed to run for the time taken by MOGASVM, they are not able to improve their clustering results any further. The average execution times of MOGASVM for the Adult malignancy and the Brain tumor datasets are 212.76 and 81.28 seconds, respectively. The timing requirements of the proposed technique can be reduced further by using a stopping criterion based on some test of convergence of the multiobjective evolutionary process.

### Statistical Significance Test

To establish that MOGASVM is significantly superior to the other algorithms, a statistical significance test called t-test has been conducted at 5% significance level. Ten groups, corresponding to the ten algorithms (1. MOGASVM, 2. K-means, 3. EM, 4. SGA, 5. average linkage, 6. SOM, 7. SiMM-TS, 8. CSPA, 9. HGPA, 10. MCLA) have been created for each dataset. Each group consists of the 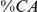 index scores produced by 50 consecutive runs of the corresponding algorithm.

As is evident from the Tables 2,3,4, the average values of the 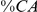 scores for MOGASVM are better than those for the other algorithms. To establish that this goodness is statistically significant, Table 5 reports the *P-values* produced by t-test for comparison of two groups (the group corresponding to MOGASVM and a group corresponding to some other algorithm) at a time. As a null hypothesis, it is assumed that there is no significant difference in the mean values of the two groups. Whereas, the alternative hypothesis is that there is significant difference in the mean values of the two groups. All the *P-values* reported in the table are less than 0.05 (5% significance level). This is a strong evidence against the null hypothesis, indicating that the better mean values of the 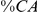 index produced by MOGASVM are statistically significant and have not occurred by chance.

**Table 5:** The *P-values* produced by t-test comparing MOGASVM with the other algorithms.

	P-values								
datasets	(comparing mean values of %CA index of MOGASVM with other algorithms)								
	K-means	EM	SGA	Avg. Link	SOM	SiMM-TS	CSPA	HGPA	MCLA
SRBCT	3.1E-07	2.17E-07	2.41E-03	1.08E-06	6.5E-05	5.32E-03	3.23E-04	6.38E-06	2.94E-04
Adult malignancy	2.21E-05	1.67E-08	3.4E-05	4.52E-12	1.44E-04	2.53E-03	7.2E-04	2.3E-06	1.4E-04
Brain tumor	3.42E-05	7.43E-08	5.8E-05	2.7E-07	2.1E-05	1.4E-04	8.92E-05	6.2E-06	9.3E-05

### Gene Markers for the SRBCT Dataset

**Figure 4:**
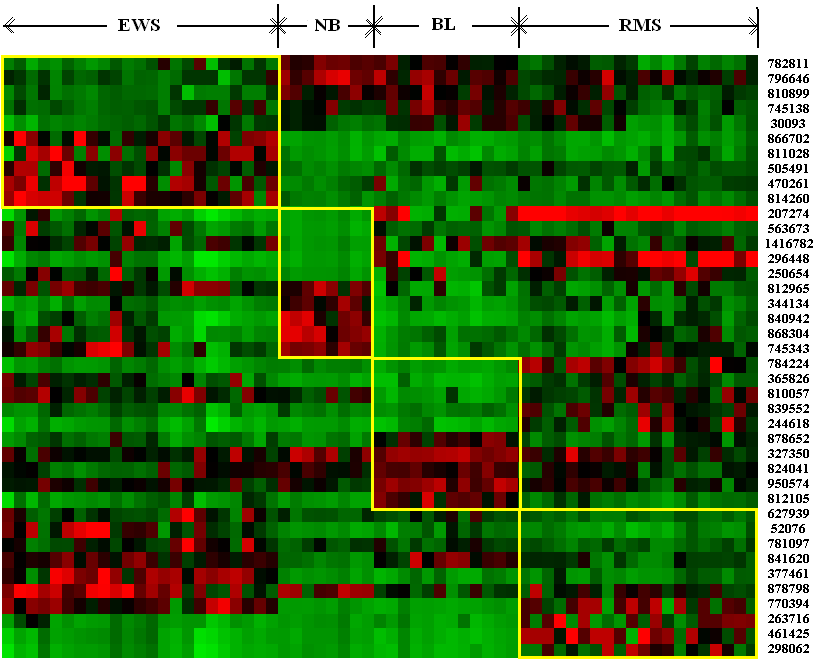
The heatmap of the expression levels of the most frequently selected top 10 gene markers for each tumor subtype in the SRBCT data. Red/green represents up/down regulation relative to black. Each subgroup is in a yellow box to identify its samples and the distinguishing gene markers. The image clone IDs of the marker genes are also shown on the right side of the genes.

In Figure 4 we have shown the heatmap of the gene versus sample matrix of the SRBCT dataset, where the rows correspond to the most frequently selected top 10 genes in terms of SNR statistic scores for each tumor subtype depicted in the columns. Thus there are a total of 40 rows, corresponding to the 40 gene markers, 10 for each of the four classes. The cells of the heatmap represent the expression levels of the genes in terms of colors. The shades of red represent high expression levels, the shades of green represent low expression levels and the colors towards black represent the absence of differential expression values. It is evident from the figure that the gene markers for each tumor subtypes have either high expression values (up-regulated) or low expression values (down-regulated) over all the samples of the respective tumor class. In Tables 6,7,8,9, we have reported the top 10 gene markers along with their description and up/down regulation states for the EWS, NB, BL and RMS tumor classes, respectively. Also the frequency of selection of each gene over 50 runs of MOGASVM is reported. For the EWS class, the genes 782811 (HMGA1), 796646 (ODC1), 810899 (CKS1B), 745138 (TUBA3D) and 30093 (RANBP1) are down-regulated and the genes 866702 (PTPN13), 811028 (TMEM49), 505491 (PTTG1IP), 470261 (SMA4) and 814260 (KDSR) are up-regulated. It is interesting to observe that these genes behave almost oppositely in the remaining tumor classes (Figure 4). For the NB class, the genes 207274 (IGF2), 563673 (ALDH7A1), 1416782 (CKB), 296448 (IGF2) and 250654 (SPARC) are down-regulated and the genes 812965 (MYC), 344134 (IGLL1), 840942 (HLA-DPB1), 868304 (ACTA2) and 745343 (REG1A) are up-regulated. For the BL class, the down-regulated genes are 784224 (FGFR4), 365826 (GAS1), 810057 (CSDA), 839552 (NCOA1) and 244618 (FNDC5), whereas the up-regulated genes are 878652 (PCOLCE), 327350 (HNRNPA2B1), 824041 (SFRS9), 950574 (H3F3B) and 812105 (MLLT11). Lastly, for the RMS class, the down-regulated and up-regulated genes are 627939 (CSRP3), 52076 (OLFM1), 781097 (RTN3), 841620 (DPYSL2), 377461 (CAV1) and 878798 (B2M), 770394 (FCGRT), 263716 (COLGA1), 461425 (MYL4), 298062 (TNNT2), respectively.

**Table 6:** The gene markers in the SRBCT data for the EWS class, their Image IDs, symbols, selection frequencies, descriptions and up/down regulation natures.

Gene Image ID	Symbol	Frequency %	Description	Up/Down
782811	HMGA1	100	high-mobility group (nonhistone chromosomal)	Down
			protein isoforms I and Y	
796646	ODC1	100	ornithine decarboxylase 1	Down
810899	CKS1B	96	CDC28 protein kinase regulatory subunit 1B	Down
745138	TUBA3D	96	tubulin, alpha	Down
30093	RANBP1	90	RAN binding protein	Down
866702	PTPN13	100	protein tyrosine phosphatase, non-receptor type 13	Up
			(APO-1/CD95 (Fas)-associated phosphatase)	
811028	TMEM49	98	transmembrane protein 49	Up
505491	PTTG1IP	98	pituitary tumor-transforming 1 interacting protein	Up
470261	SMA4	94	glucuronidase, beta pseudogene	Up
814260	KDSR	92	3-ketodihydrosphingosine reductase	Up

**Table 7:** The gene markers in the SRBCT data for the NB class, their Image IDs, symbols, selection frequencies, descriptions and up/down regulation natures.

Gene Image ID	Symbol	Frequency %	Description	Up/Down
207274	IGF2	100	Human DNA for insulin-like growth factor II (IGF-2);	Down
			exon 7 and additional ORF	
563673	ALDH7A1	100	aldehyde dehydrogenase 7 family, member A1	Down
1416782	CKB	100	creatine kinase, brain	Down
296448	IGF2	96	insulin-like growth factor 2 (somatomedin A)	Down
250654	SPARC	92	secreted protein, acidic, cysteine-rich (osteonectin)	Down
812965	MYC	100	v-myc avian myelocytomatosis viral oncogene homolog	Up
344134	IGLL1	100	immunoglobulin lambda-like polypeptide	Up
840942	HLA-DPB1	94	major histocompatibility complex, class II, DP beta	Up
868304	ACTA2	94	actin, alpha 2, smooth muscle, aorta	Up
745343	REG1A	90	regenerating islet-derived 1 alpha	Up
			(pancreatic stone protein, pancreatic thread protein)	

**Table 8:** The gene markers in the SRBCT data for the BL class, their Image IDs, symbols, selection frequencies, descriptions and up/down regulation natures.

Gene Image ID	Symbol	Frequency %	Description	Up/Down
784224	FGFR4	100	fibroblast growth factor receptor	Down
365826	GAS1	98	growth arrest-specific	Down
810057	CSDA	98	cold shock domain protein A	Down
839552	NCOA1	94	nuclear receptor coactivator	Down
244618	FNDC5	94	fibronectin type III domain containing 5	Down
878652	PCOLCE	100	procollagen C-endopeptidase enhancer	Up
327350	HNRNPA2B1	100	heterogeneous nuclear ribonucleoprotein A2/B1	Up
824041	SFRS9	100	splicing factor, arginine/serine-rich	Up
950574	H3F3B	96	H3 histone, family 3B (H3.3B)	Up
812105	MLLT11	92	myeloid/lymphoid or mixed-lineage leukemia	Up
			(trithorax homolog, Drosophila); translocated to, 11	

**Table 9:** The gene markers in the SRBCT data for the RMS class, their Image IDs, symbols, selection frequencies, descriptions and up/down regulation natures.

Gene Image ID	Symbol	Frequency %	Description	Up/Down
627939	CSRP3	100	cysteine and glycine-rich protein 3	Down
			(cardiac LIM protein)	
52076	OLFM1	100	olfactomedinrelated ER localized protein	Down
781097	RTN3	100	neurotrophic tyrosine kinase, receptor-related	Down
841620	DPYSL2	98	dihydropyrimidinase-like	Down
377461	CAV1	98	caveolin 1, caveolae protein, 22kD	Down
878798	B2M	100	beta-2-microglobulin	Up
770394	FCGRT	98	Fc fragment of IgG, receptor, transporter, alpha	Up
263716	COL6A1	98	collagen, type VI, alpha	Up
461425	MYL4	96	myosin light chain 4	Up
298062	TNNT2	96	troponin T2, cardiac	Up

Among the above gene markers, many of those have already been validated to be associated with the respective cancer classes in different existing literature. For example, the gene PTPN13 has been shown to be over-expressed for EWS (thus has been treated as a marker for the EWS class) in [31]. In [32] and [33], the relation of IGF2 with neuroblastoma (NB) has been investigated and IGF2 has been found to be a good marker for the NB class. For another gene CKB, it has been stated in [34] that the cytosolic CKB is induced in a variety of tumors, including neuroblastoma. Moreover, the work in [35] reveals that the gene SPARC potently inhibits angiogenesis and significantly impairs the NB tumor growth *in vivo*. In [36], the role for 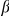
2-Microglobulin (B2M) in echovirus infection of rhabdomyosarcoma (RMS) cells has been investigated. The gene MYL4 has been shown to be over-expressed in Alveolar rhabdomyosarcoma (ARMS) class by gene expression profiling. Finally, TNNT2 has also been shown to be highly expressed for the RMS class in [37]. This discussion indicates that our approach has identified many potential gene markers that are also shown to be associated with the respective cancer types in different existing studies. Therefore, it will be interesting to conduct some biological experimentation to investigate the roles of the other marker genes selected in this work.

To look into the biological relationship among the selected gene markers, gene ontology based study has been conducted using FatiGO (http://babelomics.bioinfo.cipf.es/). The outcome of the study has been reported in Tables 10,11,12,13 for the gene markers of the four tumor classes EWS, NB, BL and RMS, respectively. Each table reports a list of GO terms (under biological process category) shared by the marker genes of the corresponding tumor class. For each GO term, the percentage of genes sharing that term among the marker genes and percentage of genes sharing that term among the whole genome have been reported. It is evident from the tables that the percentage among the gene markers is much higher than the percentage among the whole genome. This indicates that the gene markers of a particular tumor class are more involved in similar biological processes compared to the remaining genes of the genome.

**Table 10:** The significant GO terms shared by the gene markers in the SRBCT data for the EWS class. Level refers to the GO Level.

Level	GO term	Module %	Genome %
3	cellular component organization and biogenesis (GO:0016043)	50.0	18.3
4	transport (GO:0006810)	42.86	18.33
3	multicellular organismal development (GO:0007275)	25.0	15.83
3	nitrogen compound metabolic process (GO:0006807)	12.5	3.24
3	protein localization (GO:0008104)	12.5	5.28
4	carbohydrate metabolic process (GO:0005975)	14.29	3.72
4	amino acid and derivative metabolic process (GO:0006519)	14.29	2.48
6	DNA replication (GO:0006260)	14.29	1.7
6	biogenic amine metabolic process (GO:0006576)	14.29	0.52

Module % is the percentage of the genes involved in the particular GO term among the gene markers. Genome % is the percentage of genes involved in the particular GO term among the complete genome.

**Table 11:** The significant GO terms shared by the gene markers in the SRBCT data for the NB class.

Level	GO term	Module %	Genome %
3	cell proliferation (GO:0008283)	42.86	5.46
3	immune response (GO:0006955)	28.57	5.38
3	cell cycle (GO:0007049)	28.57	6.23
3	response to abiotic stimulus (GO:0009628)	14.29	1.02
3	antigen processing and presentation (GO:0019882)	14.29	0.84
3	tissue remodeling (GO:0048771)	14.29	0.75
6	regulation of progression through cell cycle (GO:0000074)	40.0	4.55
7	transmembrane receptor protein tyrosine	66.67	1.9
	kinase signaling pathway (GO:0007169)		

Level refers to the GO Level. Module % is the percentage of the genes involved in the particular GO term among the gene markers. Genome % is the percentage of genes involved in the particular GO term among the complete genome.

**Table 12:** The significant GO terms shared by the gene markers in the SRBCT data for the BL class.

Level	GO term	Module %	Genome %
3	response to stress (GO:0006950)	69.72	7.24
4	mitotic cell cycle (GO:0000278)	16.67	2.04
6	regulation of progression through cell cycle (GO:0000074)	16.67	4.45
8	S phase of mitotic cell cycle (GO:0000084)	20.0	0.14
9	RNA splicing, via transesterification reactions with bulged adenosine as nucleophile (GO:0000377)	25.0	1.83

Level refers to the GO Level. Module % is the percentage of the genes involved in the particular GO term among the gene markers. Genome % is the percentage of genes involved in the particular GO term among the complete genome.

**Table 13:** The significant GO terms shared by the gene markers in the SRBCT data for the RMS class.

Level	GO term	Module %	Genome %
3	circulation (GO:0008015)	25.0	1.07
3	antigen processing and presentation (GO:0019882)	25.0	0.84
3	multicellular organismal development (GO:0007275)	50.0	15.83
3	anatomical structure development (GO:0048856)	50.0	14.44
3	cellular developmental process (GO:0048869)	50.0	15.58
3	regulation of biological quality (GO:0065008)	25.0	4.14
3	immune response (GO:0006955)	25.0	5.38
4	endothelial cell proliferation (GO:0001935)	12.5	0.09
4	homeostatic process (GO:0042592)	25.0	2.73
4	cell differentiation (GO:0030154)	50.0	16.04
6	regulation of endothelial cell proliferation (GO:0001936)	20.0	0.09
6	cardiac inotropy (GO:0002026)	20.0	0.05
6	muscle development (GO:0007517)	40.0	1.47
6	sterol transport (GO:0015918)	20.0	0.06
6	glycerolipid metabolic process (GO:0046486)	20.0	0.19
7	negative regulation of endothelial cell proliferation (GO:0001937)	25.0	0.07
7	cholesterol transport (GO:0030301)	25.0	0.08
7	regulation of nitric oxide biosynthetic process (GO:0045428)	25.0	0.09
7	cardiac muscle development (GO:0048738)	25.0	0.05
9	protein oligomerization (GO:0051259)	66.67	1.22

Level refers to the GO Level. Module % is the percentage of the genes involved in the particular GO term among the gene markers. Genome % is the percentage of genes involved in the particular GO term among the complete genome.

For the purpose of illustration, the 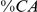 scores have been computed for the clustering solutions generated by all the algorithms on the complete preprocessed SRBCT dataset (with the initially selected 200 genes) and on the reduced dataset consisting of only the marker genes as selected using the SNR statistic. Moreover, we also tested the performance of the t-statistic as a marker gene selector. It is defined as 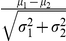, where 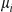 and 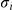 are as defined in Eqn. 13. The 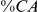 is also computed for the clustering solutions for the dataset consisting of only the marker genes selected using the t-statistic. The average 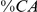 scores over 50 runs of each of the clustering algorithms for the SRBCT dataset consisting of the initially selected 200 genes, marker genes selected using the t-statistic and marker genes selected using the SNR statistic are reported in Table 14. It is evident from the table that the performance of the algorithms gets improved irrespective of the clustering algorithm used, when applied to the dataset with the identified marker genes only. Moreover, the performance of the SNR statistic is found to be better compared to that of the t-statistic. This indicates the ability of the gene markers to distinguish the different classes of samples in the datasets.

**Table 14:** The performance of the clustering algorithms on the SRBCT dataset with the initially selected 200 genes, the marker genes selected using the t-statistic and the marker genes selected using the SNR statistic.

Algorithms	%CA		
	Initially selected	Markers selected	Markers selected
	200 genes	by the t-statistic	by the SNR statistic
MOGASVM	76.6412	85.8293	90.3781
K-means	70.1903	80.3772	85.3914
EM	71.1295	82.3371	86.8934
SGA	70.8193	81.1823	86.4927
Avg. linkage	49.0527	70.2947	76.9837
SOM	71.7845	82.7845	86.9833
SiMM-TS	74.4853	84.9648	89.1397
CSPA	72.0297	83.2983	88.2286
HGPA	67.4533	77.8447	83.9824
MCLA	71.9764	83.1845	87.9411
